# The Role of Human Dicer-dsRBD in Processing Small Regulatory RNAs

**DOI:** 10.1371/journal.pone.0051829

**Published:** 2012-12-13

**Authors:** Christopher Wostenberg, Jeffrey W. Lary, Debashish Sahu, Roderico Acevedo, Kaycee A. Quarles, James L. Cole, Scott A. Showalter

**Affiliations:** 1 Department of Chemistry and Center for RNA Molecular Biology, The Pennsylvania State University, University Park, Pennsylvania, United States of America; 2 National Analytical Ultracentrifugation Facility, University of Connecticut, Storrs, Connecticut, United States of America; 3 Department of Molecular and Cell Biology and Department of Chemistry, University of Connecticut, Storrs, Connecticut, United States of America; Griffith University, Australia

## Abstract

One of the most exciting recent developments in RNA biology has been the discovery of small non-coding RNAs that affect gene expression through the RNA interference (RNAi) mechanism. Two major classes of RNAs involved in RNAi are small interfering RNA (siRNA) and microRNA (miRNA). Dicer, an RNase III enzyme, plays a central role in the RNAi pathway by cleaving precursors of both of these classes of RNAs to form mature siRNAs and miRNAs, which are then loaded into the RNA-induced silencing complex (RISC). miRNA and siRNA precursors are quite structurally distinct; miRNA precursors are short, imperfect hairpins while siRNA precursors are long, perfect duplexes. Nonetheless, Dicer is able to process both. Dicer, like the majority of RNase III enzymes, contains a dsRNA binding domain (dsRBD), but the data are sparse on the exact role this domain plays in the mechanism of Dicer binding and cleavage. To further explore the role of human Dicer-dsRBD in the RNAi pathway, we determined its binding affinity to various RNAs modeling both miRNA and siRNA precursors. Our study shows that Dicer-dsRBD is an avid binder of dsRNA, but its binding is only minimally influenced by a single-stranded – double-stranded junction caused by large terminal loops observed in miRNA precursors. Thus, the Dicer-dsRBD contributes directly to substrate binding but not to the mechanism of differentiating between pre-miRNA and pre-siRNA. In addition, NMR spin relaxation and MD simulations provide an overview of the role that dynamics contribute to the binding mechanism. We compare this current study with our previous studies of the dsRBDs from Drosha and DGCR8 to give a dynamic profile of dsRBDs in their apo-state and a mechanistic view of dsRNA binding by dsRBDs in general.

## Introduction

The past decade has seen sustained interest in the role of small regulatory RNAs, most notably microRNAs (miRNAs) and small interfering RNAs (siRNAs), in gene regulation. Both of these RNA classes function in RNA interference (RNAi) by affecting gene translation through base-pairing with messenger RNA (mRNA) via their association with Argonaute-(Ago) family proteins. [1], [2] The roles of RNAi include defense against viruses, [3], [4] regulation of development, [5] and maintenance of cellular homeostasis. [6]–[8] siRNAs can be derived either endogenously from repetitive sequences or exogenously from viral RNAs, whereas miRNAs are only endogenously transcribed. [1] Both siRNAs and miRNAs are processed by the RNase III enzyme Dicer into ∼21 nt RNAs prior to associating with Ago-family proteins, forming the RNA-induced silencing complex (RISC). [1], [2] Human Dicer processes pre-miRNA more rapidly than pre-siRNA in the absence of cofactor proteins (e.g., TRBP and PACT), [9] demonstrating that Dicer is capable of discriminating between these two classes of substrate. Dicer structures reconstructed from cryo-electron microscopy (cryo-EM) yield contrasting models for Dicer-RNA interaction and substrate selection. [10], [11] Resolving the respective mechanisms for miRNA and siRNA processing by Dicer is intriguing biochemically because of the extensive structural differences between miRNA and siRNA precursors.

**Figure 1:**
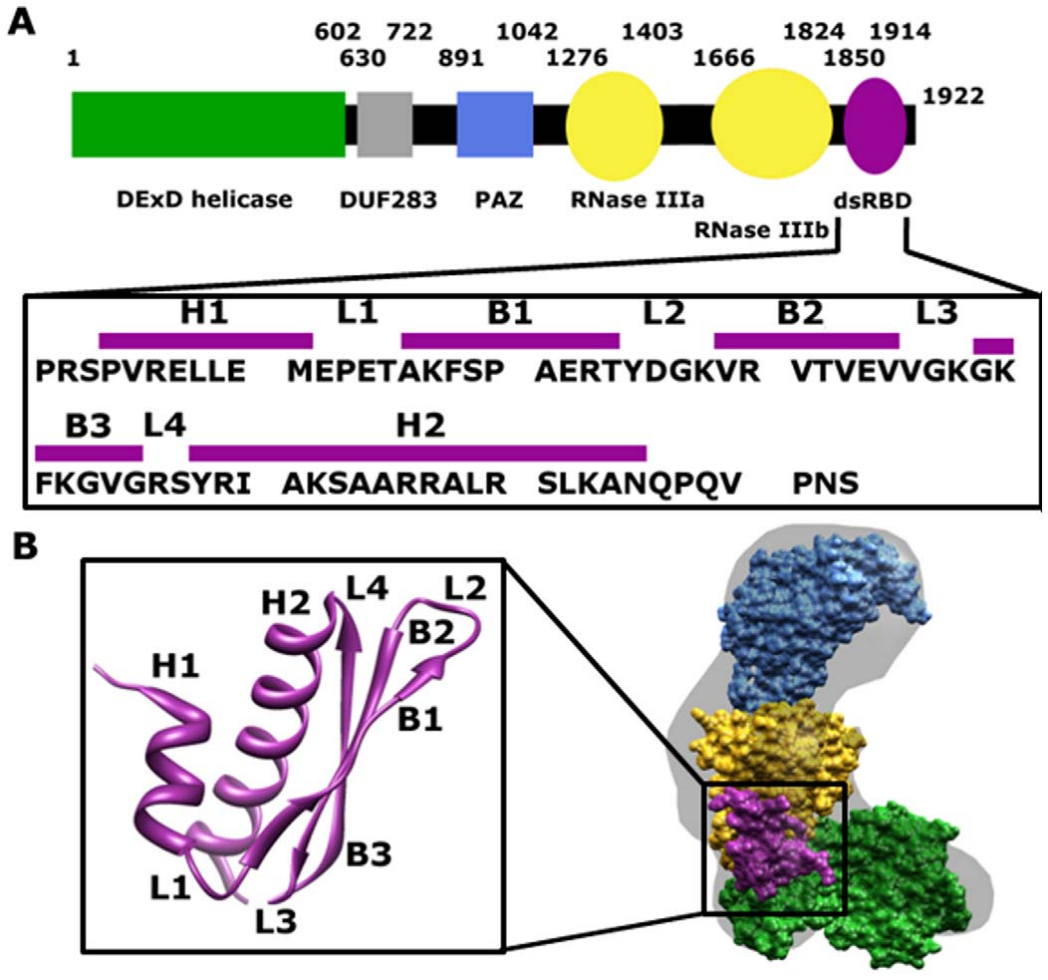
Schematic representation and structure of Dicer-dsRBD. (**A**) Schematic representation of the primary sequence of Dicer with approximate location of domains above the cartoon. The primary sequence of human Dicer-dsRBD (residues 1850 to 1922) utilized in this study is shown in the box below the schematic, with the approximate location of secondary elements shown above the sequence(H represents an α-helix, B a β-sheet, and L a loop/turn). (**B**) Cryo-EM reconstruction is shown with the surface colored gray and the individual domains colored the same as in the above schematic. The tertiary structure of the mouse Dicer-dsRBD (pdb 3C4B, residues 1833 to 1900), which is 100% identical to the human sequence (residues 1849 to 1916), is depicted in the box to the right of the cryo-EM reconstruction.

Human Dicer is a 1922 amino acid residue protein that contains a helicase domain, a domain of unknown function (DUF283), a PAZ domain on the N-terminal side of the RNase III domains, and a dsRNA binding domain (dsRBD) on the C-terminal side (Figure **1A**). [2] Most metazoan Dicers have a similar domain architecture, but much of our biochemical information is derived from studying variants found in lower organisms (e.g., *Giardia intestinalis*), which is minimally composed of the PAZ domain, a “ruler” domain that shows species variation, and two RNase III domains; this core region of Dicer is represented by the blue and yellow domains in Figure **1A**. In the minimal mechanism, the PAZ domain of Dicer binds the 3′ two-nucleotide overhang on the substrate – generated either by Drosha cleavage for miRNA or a prior round of Dicer cleavage for siRNA – and positions the catalytic sites of the intramolecular RNase III domain dimer for hydrolysis of each strand in the dsRNA, [12] resulting in cleavage to produce a new 3′ two-nucleotide overhang. [2], [13], [14] Analysis of the *Giardia* Dicer crystal structure reveals a 65 Å spacing between the PAZ domain and the RNase III catalytic sites, resulting in a roughly 25 base-pair A-form RNA product that corresponds to the length of mature siRNAs in *Giardia*. [15] Differential positioning of the “ruler” domain has been suggested as the mechanism for producing the more characteristic 21 base-pair product of human Dicer. [11].

The functional value of the helicase domain at the N-terminus of Dicers from metazoa (green in Figure **1A**) has been a mystery until very recently. *Drosophila melanogaster*, like many insects, has two Dicer paralogs tasked individually with processing pre-miRNA (Dicer-1) and pre-siRNA (Dicer-2). *Drosophila* Dicer-1 has been shown to engage the terminal loop of pre-miRNA with its helicase domain, thus establishing the correct distance from the PAZ domain for cleavage. [16] In a separate study, the helicase domains of both *Drosophila* Dicer-2 and of *Caenorhabditis elegans* Dicer-1 were shown to engage blunt-ended pre-siRNA models and establish a processive cleavage mode that is ATP-dependent. [17] These studies establish the importance of defining the functional roles of the peripheral domains found in metazoan Dicers.

The final domain missing from *Giardia* Dicer that is found in human Dicer is the C-terminal dsRBD. In most cases, dsRBDs coexist with the RNase III domains in RNase III proteins. [2], [18] The crystal structure of *Mus musculus* (mouse) Dicer-dsRBD, whose protein sequence is 100% identical to human Dicer-dsRBD, exhibits the canonical structure for dsRBDs (sequence and structure shown in Figure **1A and 1B**, respectively). [19] The cryo-EM reconstruction of human Dicer (Figure **1B**) positions the dsRBD (purple) adjacent to the RNase III domains (yellow) and on the opposite side of the “ruler” from the PAZ and platform domains (blue). [11] Proximity of the dsRBD to the RNase III domains suggests a functional role in binding and/or cleavage for the dsRBD. Deletion of human Dicer-dsRBD has been shown to dramatically reduce pre-miRNA processing efficiency. [20] Another study reveals that the Dicer-dsRBD, when present in a polypeptide also containing a portion of the RNase IIIb domain, is capable of inhibiting wild-type Dicer from binding dsRNA. [21] More recently, Doudna and coworkers have demonstrated that the dsRBD of human Dicer is required for substrate binding and cleavage in the context of an N-terminally truncated construct. [22] Importantly, the evidence for Dicer-dsRBD function from all of these studies is indirect – RNA binding by the isolated dsRBD was not demonstrated and so a specific mechanistic role for the domain could not be assigned. This point is significant, because the isolated dsRBD from human Drosha, another RNase III enzyme in the miRNA maturation pathway, is unable to bind dsRNA due to non-canonical structural features and dynamics, [23] even though the presence of the Drosha-dsRBD is required for activity. [24].

To further test the functional role of Dicer-dsRBD and establish its dsRNA binding activity, we have expressed human Dicer-dsRBD in isolation and determined its binding affinity to various RNAs modeling pre-miRNA and pre-siRNA. Previously, we have established the *in vitro* dsRNA binding affinity of both Drosha-dsRBD and the first dsRBD of Drosha’s cofactor DGCR8 using a related primary miRNA (pri-miRNA) sequence under similar conditions. [23] As in our previous study, Dicer-dsRBD binding activity is determined here by electrophoretic mobility shift assays (EMSAs), while backbone dynamics are studied with both NMR spin relaxation and MD simulations. We conclusively show that Dicer-dsRBD in isolation is able to bind dsRNA and has similar backbone dynamics to DGCR8-dsRBD1, which are distinct from those in Drosha-dsRBD. The dynamic profile of Dicer-dsRBD presented in this study will be discussed in the context of prior results from other dsRBDs. [23], [25], [26] We hypothesize a role for backbone dynamics in the binding mechanism that is consistent with the available data and testable through future experiments. In addition, we show that Dicer-dsRBD binding is only minimally influenced by the length of RNA or by the presence of a ss-ds junction caused by a large terminal loop or a ssRNA tail adjacent to the dsRNA stem. Thus, while Dicer-dsRBD is necessary for substrate binding and cleavage, it does not independently contribute to selection between pre-miRNA and pre-siRNA substrates.

## Materials and Methods

### Protein Preparation

A synthetic Dicer gene was purchased from Geneart and a Dicer-dsRBD construct (1850–1922) was PCR amplified. The PCR product was then cloned into pET47b (Novagen), which encodes a 6X His tag and a 3C protease recognition site upstream of the cloning site. Next, the plasmid was transformed into BL21 (DE3) competent cells. For NMR experiments, the cells were grown at 37°C with shaking in a liter of M9 minimal media with [^15^N] ammonium chloride as the only nitrogen source and either [^12^C]- or [^13^C] glucose as the only carbon source to produce uniformly ^15^N or uniformly ^15^N, ^13^C- labeled protein. The cells were induced with 500 µL of 1.0 M IPTG when OD_600_ was between 0.5 and 0.6, then harvested after 3.5 hrs. For EMSAs, the cells were grown in 500 mL of LB media and induced with 250 µL of 1.0 M IPTG when the OD_600_ was between 0.8 and 1.0, then harvested after 3.5 hrs. The cells were lysed by sonication at 4°C and the resulting suspension was centrifuged for 30 min at 11,500 rpm in a Beckman Coulter Allegra 25R using a TA-14-50 rotor. The clear supernatant was then passed over a Ni-NTA (Novagen) column, and the protein was eluted with imidazole (200 mM). The His tag was cleaved using 3C protease at 4°C overnight while also dialyzing away the imidazole. The content of the dialysis bag was then passed over the same Ni-NTA column, and the flow-through was collected. The protein was concentrated and buffer exchanged using an Amicon Ultra centrifugal filter device (Millipore) that contained a PES 3000 MWCO membrane. For EMSAs, the final buffer was 50 mM cacodylate, pH 6.0 and 50 mM potassium chloride, while for NMR studies the final buffer was 50 mM Tris, pH 7.5 and 100 mM KCl. The final concentration of the protein was determined by UV absorption using a ε = 2800 M ^−1^ cm ^−1^ at 278 nm.

### RNA Preparation

To test the ability of the Dicer-dsRBD to discriminate between miRNA and siRNA precursors, it was necessary that our model pre-miRNA contained a natural nucleotide sequence with a 2 nucleotide 3′ overhang. This necessitates that a different method than the standard T7 transcription be used, due to the fact that the standard procedure requires a tandem G sequence on the 5′ of the RNA to initiate transcription. Therefore, pre-mir-16-1 DNA containing the sequence for a self-cleaving hammerhead riboyzme and a T7 promotor sequence on the 5′-end and an inverted BsaI cut site at the 3′-end was purchased from Integrated DNA Technologies (IDT) as a sense strand (sequence 5′ to 3′: GTC AGA ATT CTA ATA CGA CTC ACT ATA GGG AGC GTG CTG CTA CTG ATG AGC GCG AAA GCG CGA AAG GAT TCC GAA AGG GAT CCT ATA GCA GCACG) and an antisense strand (sequence 5′ to 3′: CTG CGC ATG CGG TCT CCT TCA GCA GCA CAG TTA ATA CTG GAG ATA ATT TTA GAA TCT TAA CGC CAA TAT TTA CGT GCT GCT ATA GGA TCC C). The strands were PCR extended, then the dsDNA was inserted into pUC19 (New England Biolabs). Figure **S1** shows a schematic of this construct.

Next, the plasmid containing the construct was transformed into DH5α competent cells. The cells were grown overnight in LB media at 37°C to an OD_600_ of approximately 3.0. The cells were lysed, and the DNA was purified using a Plasmid Maxi Kit (Omega). The recovered DNA was digested with BsaI overnight at 50°C. After digestion, calf intestinal alkaline phosphatase was added and incubated another 30 minutes at 37°C to prevent self-ligation. Post-digestion, the linearized DNA was extracted with phenol-chloroform and precipitated with ethanol. The RNA was then transcribed by T7 polymerase in a 10 mL reaction mixture of 25 µg/mL linearized DNA, 40 mM Tris, pH 8.0, 25 mM MgCl_2_, 2 mM dithiothreitol, 1 mM spermidine, and 4 mM of each free NTP at 37°C for 3 hrs. Transcription yielded three major products on a denaturing polyacrylamide gel: pre-mir-16-1(65nt), hammerhead riboyzme (58nt) and uncleaved RNA (123nt). The pre-mir-16-1 band was cut out of the gel and soaked overnight at 4°C in a TEN_250_ solution. The RNA was then purified from the supernatant by ethanol precipitation and quantified by UV-Vis absorption; using ε = 666,700 M^−1 ^cm^−1^ at 260 nm.

DNA for the top and bottom strands for the 33 bp (top strand sequence 5′ to 3′: GGA TAT TTA CGT GCT GCT AAG GCA CTG CTG ACC TAT AGT GAG TCG TAT TAA TTT C, bottom strand sequence 5′ to 3′: GGT CAG CAG TGC CTT AGC AGC ACG TAA ATA TCC TAT AGT GAG TCG TAT TAA TTT C) and 44 bp (top strand sequence 5′ to 3′: GGT CTT AAC GCC AAT ATT TAC GTG CTG CTA AGG CAC TGC TGA CCT ATA GTG AGT CGT ATT AAT TTC, bottom strand sequence 5′ to 3′: GGT CAG CAG TGC CTT AGC AGC ACG TAA ATA TTG GCG TTA AGA CCT ATA GTG AGT CGT ATT AAT TTC) duplex RNAs were purchased from IDT containing a T7 promoter site on the 3′-end (5′ to 3′: TAT AGT GAG TCG TAT TAA TTT C). Also, DNA complementary to the T7 promoter site (5′ to 3′: GAA ATT AAT ACG ACT CAC TAT A) was purchased from IDT and annealed to the above DNAs to promote T7 transcription in a hemi-duplex method using the similar conditions as pre-mir-16-1. The ssRNAs were purified similar to the pre-mir-16-1. The RNAs were quantified by UV-Vis absorption using ε = 326,800 M^−1 ^cm^−1^, ε = 319,300 M^−1 ^cm^−1^, ε = 440,900 M^−1 ^cm^−1^, and ε = 422,900 M^−1 ^cm^−1^ at 260 nm for the 33 bp top, 33 bp bottom, 44 bp top, and 44 bp bottom strands, respectively.

The 12 bp (top strand sequence 5′ to 3′: GUC AGC AGU GCC, bottom strand is fully complementary to top strand), 16 bp (top strand sequence 5′ to 3′: GUC AGC AGU GCC UUA G, bottom strand is fully complementary to top strand) and 22 bp (top strand sequence 5′ to 3′: GUC AGC AGU GCC UUA GCA GCA C, bottom strand is fully complementary to top strand), along with theds16-flanking top (sequence 5′ to 3′: CUC UUA UGA UAG CAA UGU CAG CAG UGC CUU AG), ds16-flanking bottom (sequence 5′ to 3′: CUA AGG CAC AGC UGA CCA CAA CCG ACA CUU CU), ds16-tetra-stable (sequence 5′ to 3′: GUC AGC AGU GCC UUA GUU CGC UAA GGC ACU GCU GAC), ds16-tetra-U (sequence 5′ to 3′: GUC AGC AGU GCC UUA GUU UUC UAA GGC ACU GCU GAC), ds16-hexa-U (sequence 5′ to 3′: GUC AGC AGU GCC UUA GUU UUU UCU AAG GCA CUG CUG AC) and ds16-octa-U (sequence 5′ to 3′: GUC AGC AGU GCC UUA GUU UUU UUU CUA AGG CAC UGC UGA C) RNAs were purchased from Dharmacon without any post synthesis purification or deprotection. The RNAs were deprotected according to the Dharmacon protocol, and the RNAs were spun down to dryness. The RNAs were resuspended in water to get a concentration of roughly 100 µM. The concentration was verified by UV-Vis using the following molar extinction coefficients at 260 nm for the 12 bp top, 12 bp bottom, 16 bp top, 16 bp bottom, 22 bp top, 22 bp bottom, ds16-flank top, ds16-flank bottom, ds16-tetra-stable, ds16-tetra-U, ds16-hexa-U and ds16-octa-U respectively: 113,900 M^−1 ^cm^−1^, 110,300 M^−1 ^cm^−1^, 156,900 M^−1 ^cm^−1^, 151,300 M^−1 ^cm^−1^, 211,500 M^−1 ^cm^−1^, 206,300 M^−1 ^cm^−1^, 318,000 M^−1 ^cm^−1^, 304,700 M^−1 ^cm^−1^, 344,200 M^−1 ^cm^−1^, 351,400 M^−1 ^cm^−1^, 370,800 M^−1 ^cm^−1^, and 385,900 M^−1 ^cm^−1^.

### Electrophoretic Mobility Shift Assay

The RNA was radiolabeled using [γ-^32^P]ATP. For all duplex RNAs, the radiolabeled top-strand RNA was mixed with a 20-fold molar excess of cold bottom strand and purified as a duplex from an 8% native gel. Prior to mixing with protein, the pre-mir-16-1 was renatured at 95°C for 1.5 min. and 4°C for 5 min. The binding reactions incubated at room temperature for 30 min. to ensure full equilibration in the presence of 50 mM Tris-HCl, pH 7.5, 200 mM sodium chloride, 5% glycerol, 0.1 mg/mL Bovine Serum Albumin, 1 mM dithiothreitol, and 0.1 mg/mL herring sperm DNA to prevent the complex from sticking in the wells. Subsequently, the binding reactions were run on a 0.25X TBE, 10% acrylamide gel at 12 V cm^−1^ at 4°C for 3 hrs, with each lane containing 20 µCi.

**Figure 2:**
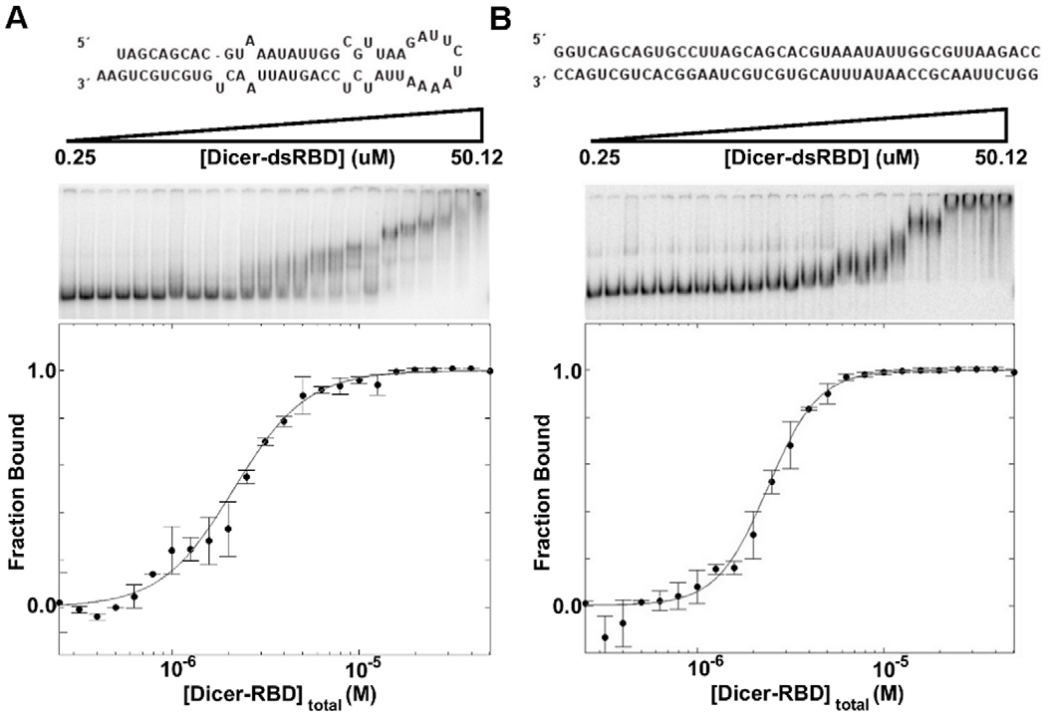
EMSA of pre-mir-16-1 and ds44 binding by Dicer-dsRBD. EMSA of Dicer-dsRBD binding (**A**) pre-mir-16-1 with a K_d_ = 2.2±0.1 µM and (**B**) ds44 with a K_d_ = 2.4±0.1 µM. The predicted secondary structures of the RNAs are shown above the representative gels, which were run with varying Dicer-dsRBD concentration (0.25–50.12 µM) binding to 0.125 nM RNA. Fraction bound, from the EMSA data, versus Dicer-dsRBD concentration was fitted using a generalized Hill model (gray line). Error bars in the plots of fraction bound as a function of total Dicer-dsRBD concentration represent the standard deviation from duplicate measurements.

Signals from the gels were quantified on a Typhoon-9410 imager and the resulting images were processed in ImageQuant. Boxes were drawn for both the free and bound RNA for each lane, and the signal was integrated to determine the fraction of bound RNA. The fraction bound was calculated as the intensity of all protein-bound species over the sum of the protein-bound species and the free RNA. The data points reported in the titration curves of Figures 2 and 4 represent the average fraction bound produced from two gels, with error bars representing the uncertainty in the mean to one standard deviation. The resulting fraction-bound curves were fit to the general Hill equation binding model, with data fitting being performed using the Levenberg-Marquardt model as implemented in Matlab (MathWorks).

### Analytical Ultracentrifugation

Sedimentation velocity analysis of Dicer-dsRBD binding to ds16 was performed using methods similar to those previously described. [27] Dicer-dsRBD and ds16 were buffer exchanged into 50 mM phosphate and 50 mM potassium chloride buffer, pH 6.00 using spin columns. Samples were loaded into two-channel aluminum-epon double-sector cells equipped with quartz windows. Data were collected using absorbance optics in a Beckman Coulter XL-I analytical ultracentrifuge. Conditions: rotor speed, 50,000 rpm; temperature, 20°C; wavelength, 260 nm. Normalized g(s*) distributions were calculated using DCDT+. [28] Association constants were determined by global analysis using SEDANAL. [29].

### NMR Methods

Standard triple resonance NMR techniques [30], [31] were used to assign the backbone resonances of apo-Dicer-dsRBD on Bruker Avance III 500 MHz spectrometer (chemical shifts are reported in Table **S1**). The spin relaxation experiments were performed on Bruker Avance III 500 MHz and 600 MHz spectrometers using standard ^15^N relaxation methods. [32], [33] All spectrometers were equipped with TCI cryoprobes for maximum sensitivity and the experiments were performed at 25°C. Spectra were processed in NMRpipe and analyzed with SPARKY (SPARKY3.113; T.D. Goddard and D. G. Kneller, University of California, San Francisco, CA). Data were analyzed in Matlab.

Titration of ds33 double-stranded RNA into ^15^N-Dicer-dsRBD was monitored using standard ^15^N HSQC experiments carried out on a Bruker Avance III 600 MHz NMR spectrometer equipped with a TCI Cryoprobe, with the sample temperature maintained at 25°C. Initially, ^15^N-labeled Dicer-dsRBD was bound to ds33-RNA (with natural isotope abundances) under dilute conditions to avoid aggregation and the resulting mixture was centrifuged in an Amicon Ultra to final concentrations of 190 µM and 380 µM respectively. Subsequently, the sample was buffer exchanged into NMR buffer (50 mM Tris HCl pH 7.5, 100 mM KCl and 10% D_2_O). This sample produced the ^15^N HSQC corresponding to the data point with a 2.0∶1.0 molar ratio of ds33-RNA to Dicer-dsRBD. A separate NMR sample was made with 190 µM of ^15^N-labeled Dicer-dsRBD in NMR buffer, corresponding to the apo sample. The titration experiments were carried out using this sample with addition of appropriate amounts of Dicer-dsRBD and ds33-RNA from the previous 2.0∶1.0 molar ratio sample to obtain NMR samples corresponding to ds33-RNA:Dicer-dsRBD molar ratios of 0.01∶1.0, 0.02∶1.0, 0.05∶1.0, and 1.0∶1.0; identical ^15^N HSQC spectra were acquired for each molar ratio.

### Model-free Analysis

Lipari-Szabo model-free fitting was performed using the program ModelFree 4.20, [34] with diffusion tensor fitting performed using the quadric method. [35], [36] The coordinates from the crystal structure of Dicer (3C4B) were used as a structural reference for diffusion tensor determination. T_1_, T_2,_ NOE data were fit to a model including axially symmetric global diffusion parameters with *S^2^*and *τ_int_* (model 2), except Asp-1875 which required a R_ex_ term (model 3).

### Molecular Dynamics Simulations

MD trajectories were run in the AMBER 11.0 software package [37] using the ff99SB [38], [39] force field. Simulations were carried out in explicit solvent represented by the SPC water model [40] under particle mesh Ewald periodic boundary conditions. [41] Dicer-dsRBD MD simulations were run as previously reported [42] using the crystal structure (3C4B, residues 1833–1900 of the mouse sequence corresponding with 1849–1916 of the human sequence). Nine chloride counterions were added to neutralize the net positive charge on the protein, and then the resulting system was solvated such that no solute atom was within 10 Å of a box edge, requiring 7031 water molecules. The starting configuration was energy minimized as previously reported. [43] Following the initial equilibration period, 250 ns of dynamics were run in an isothermal – isobaric (NPT) simulation. Snapshots from the trajectory were stored to disk every 1.0 ps. The analysis of the trajectory was done in AMBER using the ptraj program. [37] Molecular graphics images were created using the UCSF Chimera package. [44] Additional analysis and visualization was accomplished in Matlab. MD derived order parameters were obtained by using iRED analysis of MD trajectories averaged over 5 ns windows, as previously reported. [43], [45].

## Results

### Binding of Dicer-dsRBD to Pre-miRNA and Perfect Duplex RNAs

Electrophoretic mobility shift assays (EMSAs) were used to monitor the binding activity of human Dicer-dsRBD (1850–1922) in isolation. The initial study was done with pre-mir-16-1, because it represents a dsRNA that Dicer would encounter in the cell and it correlates with previous work done with pri-mir-16-1 by our group and others. [23], [46]–[49] Dicer-dsRBD is able to bind pre-miRNA with a K_d_ = 2.2±0.1 µM (Figure **2** and Table **1**), when fit to a general Hill equation binding model, as used in other binding studies. [23], [50] Dicer-dsRBD binds pre-mir-16-1 more tightly than the first dsRBD of DGCR8 in isolation binds pri-mir-16-1 (K_d_ = 9.4±0.4 µM); Dicer-dsRBD also binds pre-mir-16-1 slightly more tightly than DGCR8-Core, which contains two dsRBDs in tandem, does to pri-mir-16-1 (K_d_ = 3.7±0.1 µM). [23].

**Table 1:** Binding Affinity of Dicer-dsRBD in Isolation for Various Length RNA Constructs by EMSA.

RNA Construct	Dissociation Constant(K_d_, µM)	Hill Coefficient(n)
pre-mir-16-1	2.2±0.1	2.2±0.1
ds44	2.4±0.1	3.2±0.1
ds33	4.9±0.1	2.8±0.1
ds22	6.5±0.1	3.4±0.2
ds16	8.9±0.1	3.4±0.2
ds12	15.9±0.1	3.8±0.1

Given the dual role of Dicer in the cell, we also wanted to determine if Dicer-dsRBD is able to discriminate between pre-miRNA and pre-siRNA. To test the ability of Dicer-dsRBD to bind perfect Watson-Crick base-paired dsRNA, which is a typical structure found in siRNA precursors, we designed a 44 bp RNA, with sequence based on pri-mir-16-1. One strand of our pre-siRNA model starts from the ss-ds junction near the 5′ end of pri-mir-16-1 and continues with the wild-type sequence for 44 nucleotides. The partner strand in the duplex was designed to be the exact Watson-Crick complement of the pri-mir-16-1 derived strand (Figure **2B**, top). Dicer-dsRBD has a similar binding affinity for this construct as for pre-mir-16-1, K_d_ = 2.4±0.1 µM (Figure **2**, Table **1**). Therefore, based on this preliminary data, Dicer-dsRBD is unable to distinguish between precursors of miRNAs and those of siRNAs.

Next, we desired to establish the minimum length of duplex RNA bound efficiently by Dicer-dsRBD. Therefore, we designed four more perfect duplex RNAs of various lengths, all based on pri-mir-16-1. This was done by starting with the ds44 and consecutively removing one turn of A-form helix (∼11 bp) from the 3′-end with respect to the top strand (i.e., ds33, ds22, and ds12). Additionally, we designed a 16 base-pair perfect duplex in the same manner because it has been reported that many dsRBDs span 16 bp of A-form helix dsRNA in complex. [18], [51] In the majority of cases, dsRBDs have shown no preference for RNA sequence [18], [52] (an exception being ADAR2 [53]), so the affinity for these constructs should be based primarily on length and not sequence differences. As the length of the dsRNA is decreased, the binding affinity decreases monotonically with all the dsRNAs binding in the lower micro-molar range (Table **1**; representative gels are shown in Figure **S2**). Dicer-dsRBD binds well to 16 base-pair dsRNA, which represents a canonical minimal binding site. [18] Binding to dsRNA as short as 12 base-pairs has been observed for other dsRBDs in the past (e.g., Staufen [26]); we find Dicer-dsRBD is also able to bind 12 base-pair duplexes. Note that no large decrease in binding affinity is observed as the RNA is changed from ds33 to ds22, which is significant because ds22 represents the approximate length of a human Dicer cleavage product for both siRNA and miRNA precursors. Therefore, Dicer-dsRBD affinity alone is not sufficient to discriminate between reactant and product of the enzymatic reaction.

### Binding of Dicer-dsRBD to ds16 Utilizing Analytical Ultracentrifugation

**Figure 3:**
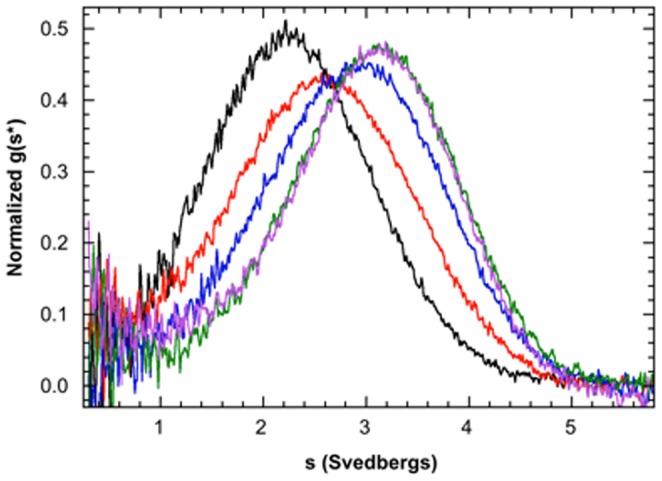
Sedimentation velocity analysis of Dicer-dsRBD binding to ds16. Plots of normalized g(s*) distributions for 2.0 µM ds16 alone (black) and 2.0 µM ds16 plus 4.3 µM (red), 8.5 µM (blue), 21.4 µM (green), and 42.7 µM (lavender) Dicer-dsRBD. The distributions are normalized by area. The shift in the peak position from 2.3 S for the RNA alone to 3.3 S for the complex corresponds to a one-to-one binding stoichiometry. The data were globally analyzed using a one-to-one binding model to yield a best fit K_d_ = 5.4±0.7 µM, which agrees well with the EMSA data.

One drawback to the EMSA method is that it is not possible to unambiguously assign binding stoichiometries. [54] The observed Hill coefficients between two and four (Table **1**) imply that multiple copies of Dicer-dsRBD bind a single RNA in a positively cooperative manner. While this seems possible for the longer dsRNAs, it seems unlikely that multiple dsRBDs can bind ds16 and ds12. The likelihood that Dicer-dsRBD encounters a lattice of overlapping binding sites, particularly when binding to the longer dsRNAs, [55]–[57] further complicates interpretation of the Hill coefficient. For this reason, we are disinclined to interpret the Hill coefficients as a biologically meaningful fitting parameter. In order to determine the exact stoichiometric ratio of protein to RNA in a saturated complex, sedimentation velocity analytical ultracentrifugation was performed using ds16 as the model RNA for the study. The data were first analyzed by the time derivative method [58] to determine the qualitative behavior of the system and to define the correct association model. Figure **3** shows normalized g(s*) distributions for a titration of ds16 with Dicer-dsRBD. The peak of the distribution shifts to the right from ∼2.3 S for ds16 to ∼3.3 S when Dicer-dsRBD binds, due to formation of a protein-RNA complex. The magnitude of this shift is consistent with binding of a single Dicer-dsRBD to the RNA. The sedimentation velocity data were globally analyzed using a 1∶1 binding model, providing a best-fit K_d_ = 5.4±0.7 µM, which agrees well with the EMSA binding affinity of K_d_ = 8.9±0.1 µM, especially when the small increase in monovalent salt concentration in the EMSAs is considered (see Materials and Methods). Therefore, we used ds16 as the basis for further constructs in the remainder of the EMSA study because we knew for certain that Dicer-dsRBD binds ds16 in a one-to-one fashion. Additionally, by choosing a single dsRNA stem length, the number of binding sites in the dsRNA lattice is fixed, thereby excluding variation in thermodynamically uninformative statistical factors from masking the impact of non-double-stranded structure on measured binding affinities.

### Effect of Hairpin Structure on Binding

**Figure 4:**
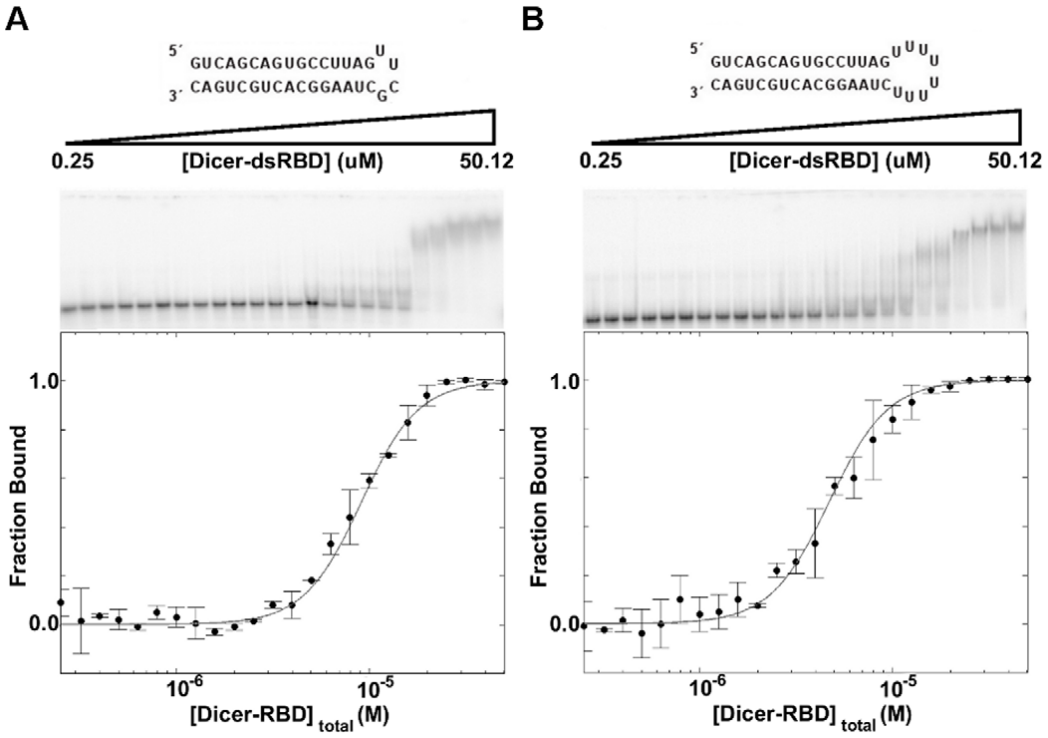
EMSA of ds16-tetra-stable and ds16-octa-U binding by Dicer-dsRBD. EMSA of Dicer-dsRBD binding (**A**) ds16-tetra-stable with a K_d_ = 9.1±0.1 µM and (**B**) ds16-octa-U with a K_d_ = 4.7±0.1 µM. The predicted secondary structures of the RNAs are shown above the representative gels, which were run with various Dicer-dsRBD concentration (0.25–50.12 µM) binding to 0.125 nM RNA. Fraction bound, from the EMSA data, versus Dicer-dsRBD concentration was fitted using a generalized Hill cooperative model (gray line). Error bars in the plots of fraction bound as a function of total Dicer-dsRBD concentration represent the standard deviation from duplicate measurements.

A major difference between pre-miRNA and pre-siRNA is that pre-miRNA has a hairpin structure. As stated earlier, dsRBDs generally do not recognize RNA sequences, but in some cases they are able to recognize structural features, specifically the structure of loops, as suggested by Rnt1p-dsRBD. [59], [60] To test if Dicer-dsRBD is able to discriminate between pre-miRNA and pre-siRNA based on the terminal loop structure, we designed four differently sized loop constructs attached to ds16: ds16-tetra-stable, containing the thermostable UUCG tetraloop; ds16-tetra-U, containing a poly-U tetraloop; ds16-hexa-U, containing a poly-U hexaloop; and ds16-octa-U, containing a poly-U octaloop. Poly-U loops were chosen because uracils do not stack upon each other well, thus ensuring the formation of open and dynamic loops of the desired sizes. Many miRNAs feature loops comparable in size to the highly flexible poly-U loop used here and have been confirmed to adopt highly disordered conformations through SHAPE reactivity (Quarles and Showalter, unpublished results). Dicer-dsRBD has the same affinity for the ds16 RNA with and without the thermostable UUCG tetraloop (Figure **4A** and Table **2**). It is only upon addition of the poly-U octaloop that Dicer-dsRBD binding affinity increases for the RNA, albeit the observed effect is a modest two-fold increase (Figure **4B** and Table **2**).

**Table 2:** Effect of Hairpin Structure on the Binding Affinity of Dicer-dsRBD for dsRNA.

RNA Construct	Dissociation Constant(K_d_, µM)	Hill Coefficient(n)
ds16-tetra-stable	9.1±0.1	2.9±0.1
ds16-tetra-U	10.7±0.1	4.6±0.3
ds16-hexa-U	8.9±0.1	3.8±0.1
ds16-octa-U	4.7±0.1	2.8±0.1
ds16-flank	4.6±0.1	2.5±0.1

It is possible that Dicer-dsRBD is recognizing the ss-ds junction caused by the large poly-U octaloop and not the structure of the loop itself. Therefore, ds16-flank was designed, where 16 nucleotides were attached to the top strand on the 5′-end and 16 nucleotides were attached to the bottom strand on the 3′-end, with sequences chosen such that the nucleotides would not form base-pairs (see Methods), therefore creating ssRNA tails on the ds16 construct. A similar binding affinity was observed for ds16-flank as for ds16-octa-U binding by Dicer-dsRBD (Table **2**; representative gels for all constructs are shown in Figure **S3**). Together, these data suggest that Dicer-dsRBD binding is minimally influenced by the presence of a ss-ds junction created by either a large terminal loop or a ssRNA tail. In the context of the whole protein, Dicer-dsRBD potentially works in collaboration with the PAZ domain to recognize the two ends of a pre-miRNA, thus facilitating correct positioning of the RNase III domains for cleavage of the dsRNA.

### Binding of Dicer-dsRBD to dsRNA Utilizing NMR Spectroscopy

**Figure 5:**
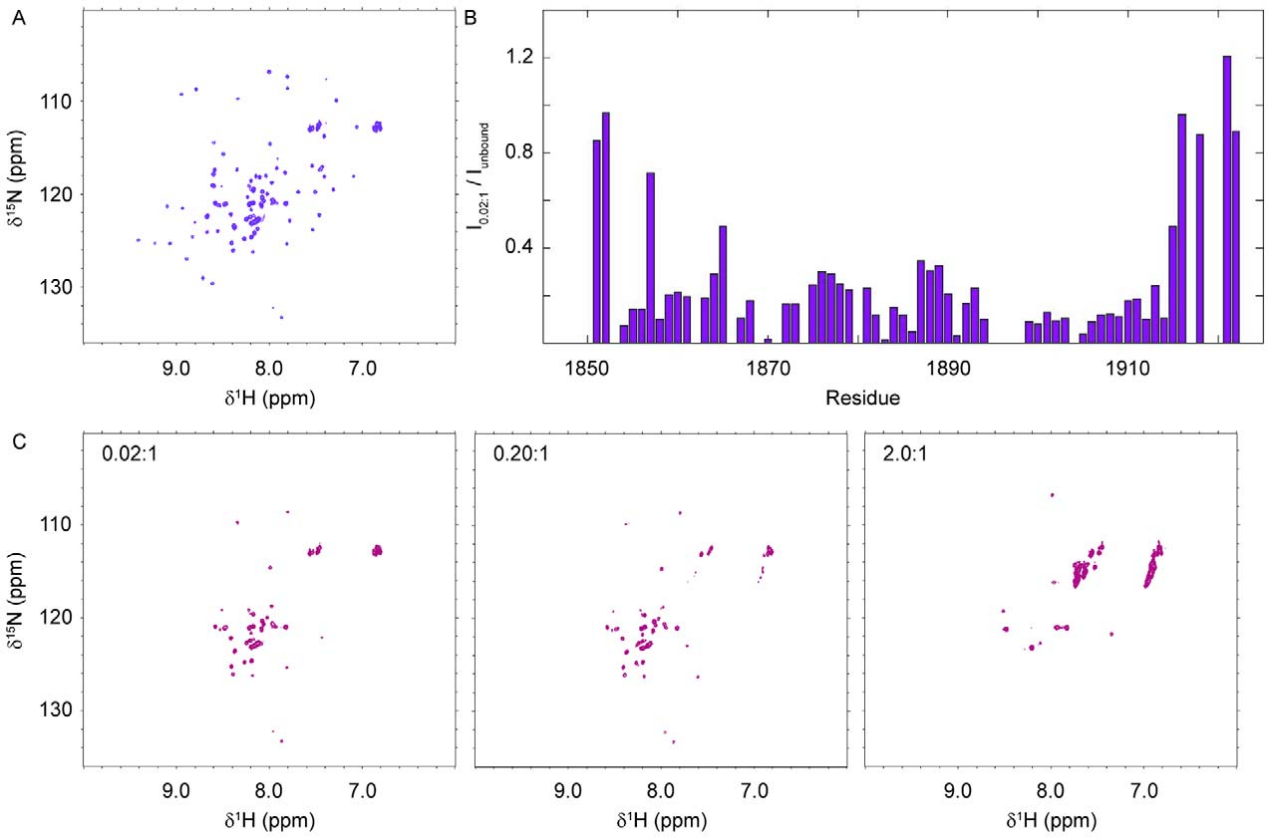
NMR titration of Dicer-dsRBD with ds33. Representative ^15^N-HSQC spectra of Dicer-dsRBD collected in the unbound state and in the presence of ds33. (A) Reference spectrum of apo-Dicer-dsRBD. (B) Ratio of individual peak intensities in the presence of 0.02∶1 mole ratio ds33:Dicer-dsRBD to those recorded under identical conditions in the apo-state spectrum displayed in (A). (C) Representative spectra from the ds33 titration showing the data points with mole ratios of 0.02∶1, 0.20∶1, and 2.0∶1 ds33:Dicer-dsRBD as labeled. All spectra were collected at 25°C in the presence of 100 mM KCl on a spectrometer operating at 600 MHz field strength.

NMR studies of Dicer-dsRBD in isolation were initiated by running a ^15^N-HSQC, which showed good dispersion (Figure **5A**). Next, both dynamic light scattering (DLS) and NMR diffusion measurements of Dicer-dsRBD confirmed that the construct was a mono-disperse monomer at NMR concentration (data not shown). These data allowed us to proceed and make complete backbone assignments for the isolated Dicer-dsRBD. Backbone resonances of Dicer-dsRBD in the apo-state were assigned using standard triple resonance NMR techniques [30], [31] on a Bruker Avance III 500 MHz spectrometer (chemical shifts are reported in Table **S1**).

Next, we attempted to find conditions in which to form a complex between Dicer-dsRBD and one of our RNA constructs, producing NMR spectra suitable for analysis. The ability to assign the resonances of an unbound protein state does not necessarily translate directly into the ability to study a complex involving the protein, because the exchange dynamics of the complex can reduce the spectral quality; for non-specific protein-RNA complexes, unfavorable exchange dynamics are almost always encountered, [61] and complexes of Dicer-dsRBD with dsRNA proved to be no exception. We first sought to produce a complex between Dicer-dsRBD and ds16, because AUC had shown this complex to form with a 1∶1 stoichiometry. Spectral quality was too poor to progress for all screened monovalent salt concentrations (100–300 mM) and temperatures (10°C–40°C). Additionally, we found that when the monovalent salt concentration was less than 100 mM, Dicer-dsRBD precipitated at NMR concentrations. In contrast, spectra of suitable quality for qualitative analysis were achieved upon titration of ds33 into Dicer-dsRBD NMR samples containing 100 mM KCl and maintained at 25°C. Representative ^15^N-HSQC spectra recorded with ds33:Dicer-dsRBD mole ratios of 0.02∶1.0, 0.2∶1.0, and 2.0∶1.0 are shown in Figure **5C**. Given that complete assignments are available for apo-Dicer-dsRBD, we next attempted to map the dsRNA binding surface by comparing the peak intensities of Dicer-dsRBD in the presence of 0.02∶1.0 mole ratio ds33:Dicer-dsRBD with the intensities of the same peaks in the apo-Dicer-dsRBD spectrum. As seen in Figure **5B**, the entire dsRBD enters intermediate exchange even at very low mole ratios, preventing a more detailed assignment of the binding surface. The high intensity tail peaks in the bound sample serve as control data points showing that spectral quality overall was equally high in both states; intensity losses in the dsRBD are directly attributable to exchange broadening induced by binding. The high intensity ratios for the disordered tails of the dsRBD provide evidence that they do not participate directly in the binding event.

### NMR Spin Relaxation

**Figure 6:**
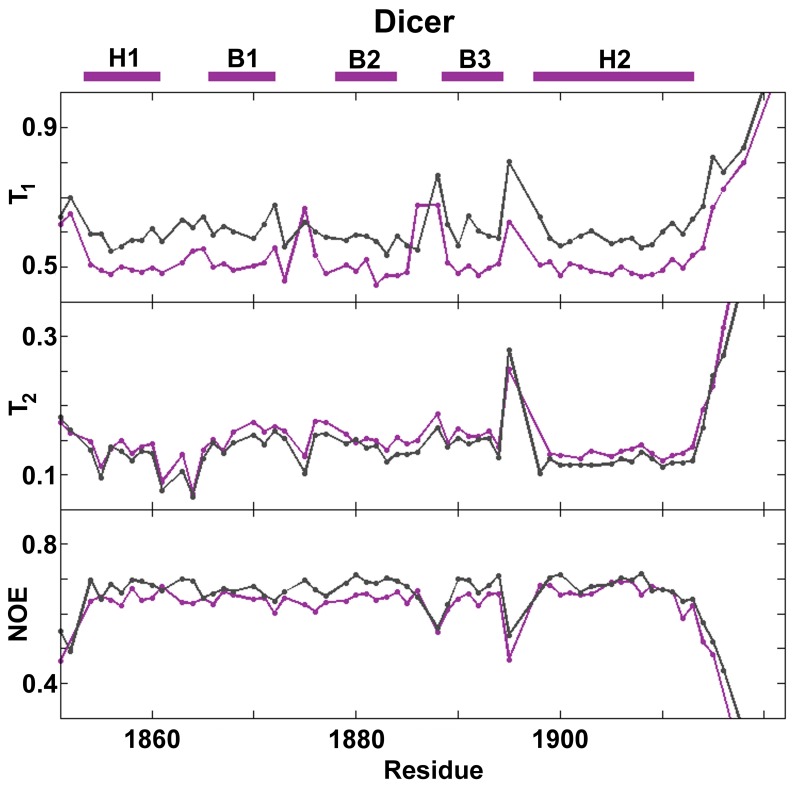
^15^N spin relaxation experiments of Dicer-dsRBD. ^15^N spin relaxation data for Dicer-dsRBD collected at 500 MHz (purple) and 600 MHz (gray) shows that the most dynamic regions of Dicer-dsRBD on the picosecond to nanosecond timescale are the loops, most notably loop 4. The secondary elements are represented as purple bars above the plot.

Apo-Dicer-dsRBD spin relaxation (^15^N T_1_, T_2_, and [^1^H]-^15^N NOE NMR) was measured at 500 MHz and 600 MHz field strength in the RNA-free state (Figure **6**). The quadric method [35], [36] used to analyze the spin relaxation data reveals that apo-Dicer-dsRBD tumbles anisotropically in solution with a D_||_/D_⊥_ = 0.51 and τ_iso_ = 6.35 ns, which agrees well with previous studies of dsRBDs from DGCR8 and Drosha (τ_iso_ = 7.20 ns and 6.29, respectively). [23] These rotational tumbling times are representative of a monomeric assembly state of a global protein domain of this size (∼ 8 kDa), agreeing with our previously mentioned DLS and NMR diffusion measurements.

**Figure 7:**
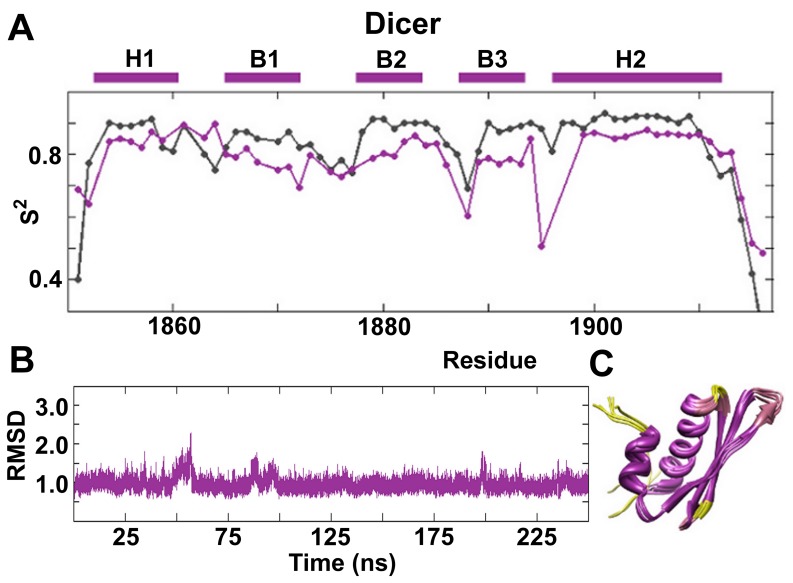
Order parameters and RMSD of Dicer-dsRBD along with PDB bundle. (**A**) Order parameters (S^2^) plotted for Dicer-dsRBD show that the most flexible regions in the protein are loops 3 and 4. Experimental data (purple) is plotted with MD predicted order parameters (gray). The secondary elements are represented as purple bars above the plot. (**B**) The overall stability of Dicer-dsRBD during the 250 ns MD simulation is demonstrated by the low average RMSD (<1.0 Å). (**C**) MD-derived ribbon bundle for Dicer-dsRBD also shows the stability of the construct. Increased flexibility, derived from the experimental order parameters, is depicted colorimetrically on the ribbon bundle as passage from purple (high order parameter) to yellow (low order parameter). The bundle was created by taking structures from the simulation every 50 ns and superimposing them to remove translational and rotational motion.

Our previous NMR data, along with that of other groups, suggest a correlation between dsRBD backbone flexibility and binding competence: flexibility is well tolerated in all loops except for loop 1, which tends not to display significant conformational dynamics in domains that bind dsRNA well. [23], [25], [26] Picosecond to nanosecond timescale backbone conformation dynamics analyzed through the generalized order parameters (S^2^) have been obtained by complete Model-free analysis of the apo-Dicer-dsRBD spin relaxation data (Figure **7A**). [62] Dicer-dsRBD shows lower order parameters in loop 3 and loop 4, indicating higher flexibility, with a minimal decrease in the order parameters for loop 1 and loop 2. Among the dsRBDs previously studied, only Staufen-dsRBD3 shows increased dynamics in loop 4 (by [^1^H]-^15^N NOE NMR spin relaxation, not S^2^). [26] The other region of increased flexibility in Staufen-dsRBD3 is loop 2, which shows increased dynamics in all of the other dsRBDs studied to date. [23], [25], [26] Dicer-dsRBD displayed elevated dynamics in this region too, albeit only slightly. Loop 2 from Dicer-dsRBD is three amino acids shorter than the canonical length, which explains its minimal increase in dynamics compared with the rest of the construct. As loop 2 directly contacts RNA in dsRBD-dsRNA complexes, the minimal dynamics of loop 2 in Dicer-dsRBD may contribute to the high binding affinity of Dicer-dsRBD for dsRNA, when compared to other dsRBDs we have studied in which loop 2 is longer and more flexible.

### Molecular Dynamics Simulations of Dicer-dsRBD

In connection with experimental data, MD simulations can provide useful dynamic information on the functional mechanisms of proteins. [63] Residues 1833 to 1900 from the crystal structure of the C-terminal region of mouse Dicer (pdb 3C4B; sequence 100% identical to residues 1849 to 1916 in human) [19] were used as the starting point for MD simulations. Root-mean-square deviation (RMSD) from the starting structure over the course of the trajectories verified that the Dicer-dsRBD was stable over the 250 ns simulation (Figure **7B**). The very low 1.0 Å RMSD seen for a large majority of the simulation is the lowest RMSD we have reported for any dsRBD in isolation, [23], [42] highlighting the high stability of the backbone of Dicer-dsRBD as compared with other dsRBDs. Further evidence of the stability of the Dicer-dsRBD comes from ribbon bundles (Figure **7C**) of the simulations overlapping well and showing no loss in secondary structure elements.

iRED analysis of the MD trajectories averaged over 5 ns windows gave computational model-free order parameters, S^2^, (Figure **7A**, gray line). [43], [45] Qualitative comparison of the computationally and experimentally derived order parameters reveals the same global trends. Note there exists an offset between the experimental and computational order parameter profiles, which is similar to Drosha-dsRBD where the experimental order parameters have a lower average than the MD derived ones. [23] Similar offsets have been observed in the past, with ff99SB sometimes producing baselines that are too high. [43] The offset is unlikely to impact the conclusions, because we do not rely on quantitative comparison between experimental and MD order parameters in this study, but rather on the trends in relative flexibility. Significantly, in all regions of the protein where experiment suggests enhanced dynamics on the ps-ns timescale, similar dynamics are also observed by MD.

**Figure 8:**
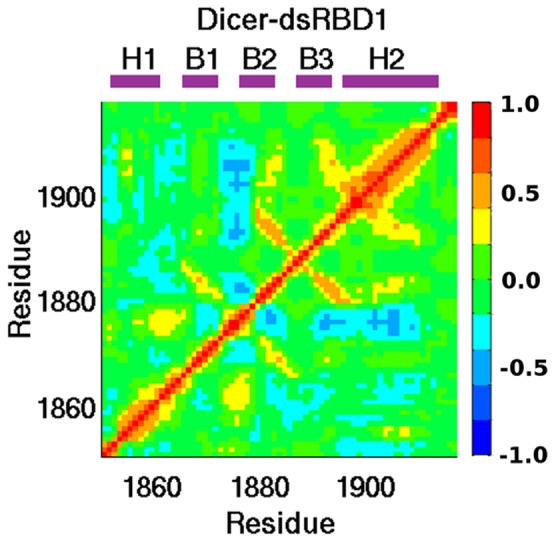
C_α_ correlations of Dicer-dsRBD. The C_α_ correlation matrix of Dicer-dsRBD reveals backbone motions. The color bar on the right shows the scale indicating strong positive correlation (red), strong negative correlation (blue), and non-correlated (green) motions. Labels above the panel indicate the location of secondary structural elements within the sequence.

In our previous study of isolated dsRBDs, we utilized principal component analysis (PCA) to investigate correlated dynamics within the dsRBDs of DGCR8 and Drosha. [23], [42] The same analysis was done with Dicer-dsRBD (Figure **8**) demonstrating that Dicer-dsRBD has similar correlated motions as DGCR8-dsRBD1 (see reference 23 Figure **7B** and reference 42 Figure **4A**). For Drosha-dsRBD, which is not able to bind RNA, we previously found that loop 1 was the most dynamic segment of the dsRBD, based on order parameters computed both from NMR data and MD trajectories; furthermore, the fluctuations of loop 1 were largely decoupled from those of the other dynamic regions of the domain, based on PCA of the MD trajectory. In contrast to the Drosha-dsRBD result, there is a major positive correlation in Dicer-dsRBD between loop 1 and loop 2, which is also observed in DGCR8-dsRBD1, while the overall dynamics of loop 1 are significantly less than those observed in Drosha-dsRBD.

## Discussion

### The Role of Dicer-dsRBD in Discriminating between siRNA and miRNA Precursors

Dicer is involved in the cleavage of siRNA precursors and miRNA precursors. Both of these types of small regulatory RNAs have different structural features that could be exploited to promote matching with appropriate pathway-specific Ago proteins by Dicer. Notably, canonical siRNA precursors are long dsRNAs composed almost exclusively of Watson-Crick base-pairs, whereas miRNA precursors are roughly 26 bp of dsRNA with internal imperfections (loops and bulges) and attached to a terminal loop. From our length study, Dicer-dsRBD shows a monotonic decrease of less than ten-fold in binding affinity as the length of dsRNA decreases from 44 to 12 base-pairs. As noted earlier, no large drop-off in binding affinity is observed going from ds33 to ds22, suggesting Dicer-dsRBD is unable to distinguish between the reactant and the product of Dicer cleavage and is therefore unlikely to promote product release.

Other than duplex length, the most striking factor for dsRNA discrimination is differences in secondary structure. Both pre-siRNA and pre-miRNA have 3′ two-nucleotide overhangs, which the PAZ domain recognizes. [2], [15] The major structural difference between pre-siRNA and pre-miRNA is the presence of the terminal loop on the pre-miRNAs. Dicer-dsRBD binding is only minimally influenced by the ss-ds junction caused by large loops, as indicated by the higher binding affinity of ds16-octa-U (K_d_ = 4.7±0.1 µM) versus the binding affinity for ds16 without a loop (K_d_ = 8.9±0.1 µM). Although this effect is small in isolation, cooperative contributions in the context of full Dicer involving the dsRBD and the helicase domain could yield substantial discriminatory value and perform a major role in the mechanism of substrate selection. This model is consistent with the recent cryo-EM reconstruction of human Dicer, in which juxtaposition of the dsRBD and helicase domains could facilitate cooperative interaction with pre-miRNA terminal loops, while the PAZ domain selects the two-nucleotide overhang on the opposite end of the “ruler domain (Figure **1B**). [11].

### Dicer-dsRBD Binding Compared to Other dsRBDs

Among the isolated or tandem dsRBDs from the miRNA processing pathways that we have studied to date, Dicer-dsRBD has the highest binding affinity. [23] If dsRNA length is the only determinant of dsRBD binding strength, DGCR8-dsRBD1 should bind pri-miRNA more tightly than Dicer-dsRBD binds pre-miRNA, because pri-miRNA is one turn of A-form RNA longer than pre-miRNA. However, this is not the case; our data illustrate that Dicer-dsRBD is better at binding dsRNA than DGCR8-dsRBD1 and in fact binds more tightly than DGCR8-Core, which contains two dsRBDs in tandem. It is intriguing to speculate that this difference in affinity may have been selected because DGCR8 and Drosha need to release the cleaved pre-miRNA for export to the cytosol by Exportin-5, whereas Dicer and its cofactors participate further in miRNA strand transfer to the RISC complex – premature product release by Dicer would be deleterious to the cell.

Protein backbone dynamics have been shown to play a vital role in target binding by a variety of proteins in multiple functional contexts. [32], [64], [65] This has inspired us to undertake a complete characterization of backbone dynamics in the set of dsRBDs found throughout the miRNA processing pathway. It must be acknowledged that our NMR studies of ds33 bound Dicer-dsRBD resulted in a uniform loss of signal intensity, compared to the unbound state. We therefore have been unable to show definitively that Dicer binds RNA with the canonical interaction surface composed of helix 1, helix 2, and loop 2, as is the general mechanism for proteins in this fold family [18]. With this limitation in mind, we have performed NMR spin relaxation on Dicer-dsRBD to establish a dynamic profile for this domain in the unbound state and discuss the potential impact of dynamics on binding, under the assumption that Dicer employs the canonical binding mode.

Dicer is unusual in that loop 2 shows only a minimal increase in flexibility when compared to loop 3 in both the experimental and computational data (Figure **6A**). In our previous work, loop 2 of DGCR8-dsRBD1 displayed dramatically lower order parameters (increased flexibility) when compared with the rest of the domain. It appears that reduced dynamics of Loop 2 in Dicer-dsRBD are not detrimental to binding.Dicer-dsRBD shows additional dynamics in loop 3 and loop 4, which were not observed in DGCR8-dsRBD1. Dicer-dsRBD is the first reported case of a dsRBD having flexibility in loop 3 of the domain. If Dicer employs the canonical binding mode, Loop 3 is on the opposite side from the RNA binding interface, so its dynamics appear unrelated to dsRNA binding unless they are correlated with the dynamics of another region of the domain that is involved in binding, thus acting in an allosteric manner. Dicer-dsRBD is not the first case of a dsRBD with increased dynamics in loop 4; based on [^1^H]-^15^N NOE NMR spin relaxation data, Staufen-dsRBD3 has increased dynamics in loop 4 in both the apo- and holo-state. [26] In summary, incorporating the Dicer-dsRBD data into our studies of dsRBD dynamics lead us to propose the following hypothesis: while ps-ns dynamics of loop 2 appear to be compatible with dsRNA binding, significant flexibility of loop 1 is not well tolerated, particularly when those dynamics are not strongly correlated with those of loop 2. Future investigations will test the validity of this proposal.

### Conclusions

In this paper, we have focused on the binding affinities between Dicer-dsRBD, in isolation from the rest of the protein, and various RNAs representing both miRNA and siRNA precursors. Our results demonstrate that the functional role of the dsRBD from human Dicer is not to discriminate between the various dsRNA substrates in the cell, but to bind all dsRNAs with roughly low micro-molar affinity. Discrimination of dsRNA substrates therefore comes from other domains within Dicer and/or from Dicer cofactors (e.g., TRBP and PACT).

Previously we have studied pri-miRNA binding and the protein dynamics of the dsRBDs involved in miRNA maturation by the Drosha-DGCR8 complex. [23] This work along with our previous studies provides a dynamic profile for the binding mechanism of dsRBDs. While the dynamics of the loops vary from one dsRBD in the apo-state to the next, one interpretation of our data is that the dynamics of loop 1 must be modest in amplitude and correlated with those of loop 2 in order to provide a well organized binding surface. It remains to be seen if a causal link between loop dynamics and binding activity can be established.

## Supporting Information

Figure S1 **Representation of transcription construct to obtain pre-mir-16-1.** Representation of the hammerhead (red) with pre-mir-16-1 (green) RNA construct used for transcription. The arrow represents the hammerhead cleavage site, which causes the release of mature pre-mir-16-1. The two cut sites (EcoR1 and Sph1) are for inserting the construct into pUC19. The inverted BsaI site is used to linearize the plasmid to avoid run on transcription.
(TIF)

Figure S2 **Representative EMSAs of Dicer-dsRBD binding to dsRNA Duplexes.** EMSA of Dicer-dsRBD binding to the indicated dsRNA constructs is shown as a representative gel (left) and the fit of fraction bound vs Dicer concentration (right). Best fit lines are to a generalized Hill model and the Kd indicated results from the fitting procedure, as described in the main text. All Watson-Crick duplex constructs used for analysis, but for which a representative gel did not appear in the main text, are represented here.
(TIF)

Figure S3 **Representative EMSAs of Dicer-dsRBD binding to Loop-terminated dsRNA Duplexes.** EMSA of Dicer-dsRBD binding to the indicated dsRNA constructs is shown as a representative gel (left) and the fit of fraction bound vs Dicer concentration (right). Best fit lines are to a generalized Hill model and the Kd indicated results from the fitting procedure, as described in the main text. All loop and tail constructs used for analysis, but for which a representative gel did not appear in the main text, are represented here.
(TIF)

Table S1 Chemical shifts (ppm) from the backbone assignment of Dicer-dsRBD.
(DOCX)
