# Supplementary figures and images for: The Role of Human Dicer-dsRBD in Processing Small Regulatory RNAs
Source: PLoS One. 2012 Dec 13;7(12):e51829. doi: 10.1371/journal.pone.0051829 (PMC3521659; doi:10.1371/journal.pone.0051829)

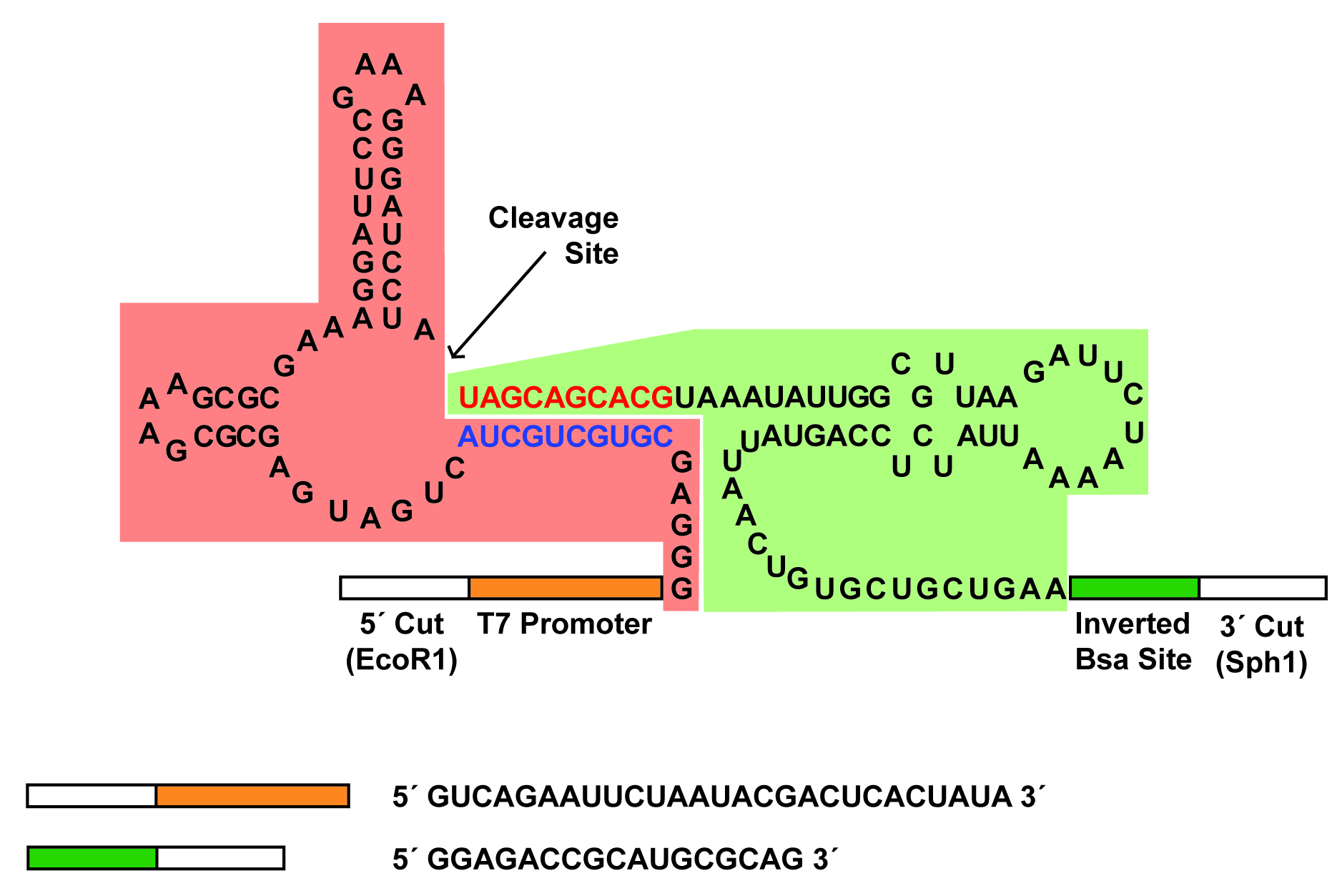

Supplement: Figure S1 — Representation of transcription construct to obtain pre-mir-16-1. Representation of the hammerhead (red) with pre-mir-16-1 (green) RNA construct used for transcription. The arrow represents the hammerhead cleavage site, which causes the release of mature pre-mir-16-1. The two cut sites (EcoR1 and Sph1) are for inserting the construct into pUC19. The inverted BsaI site is used to linearize the plasmid to avoid run on transcription. (TIF) [file pone.0051829.s001.tif]

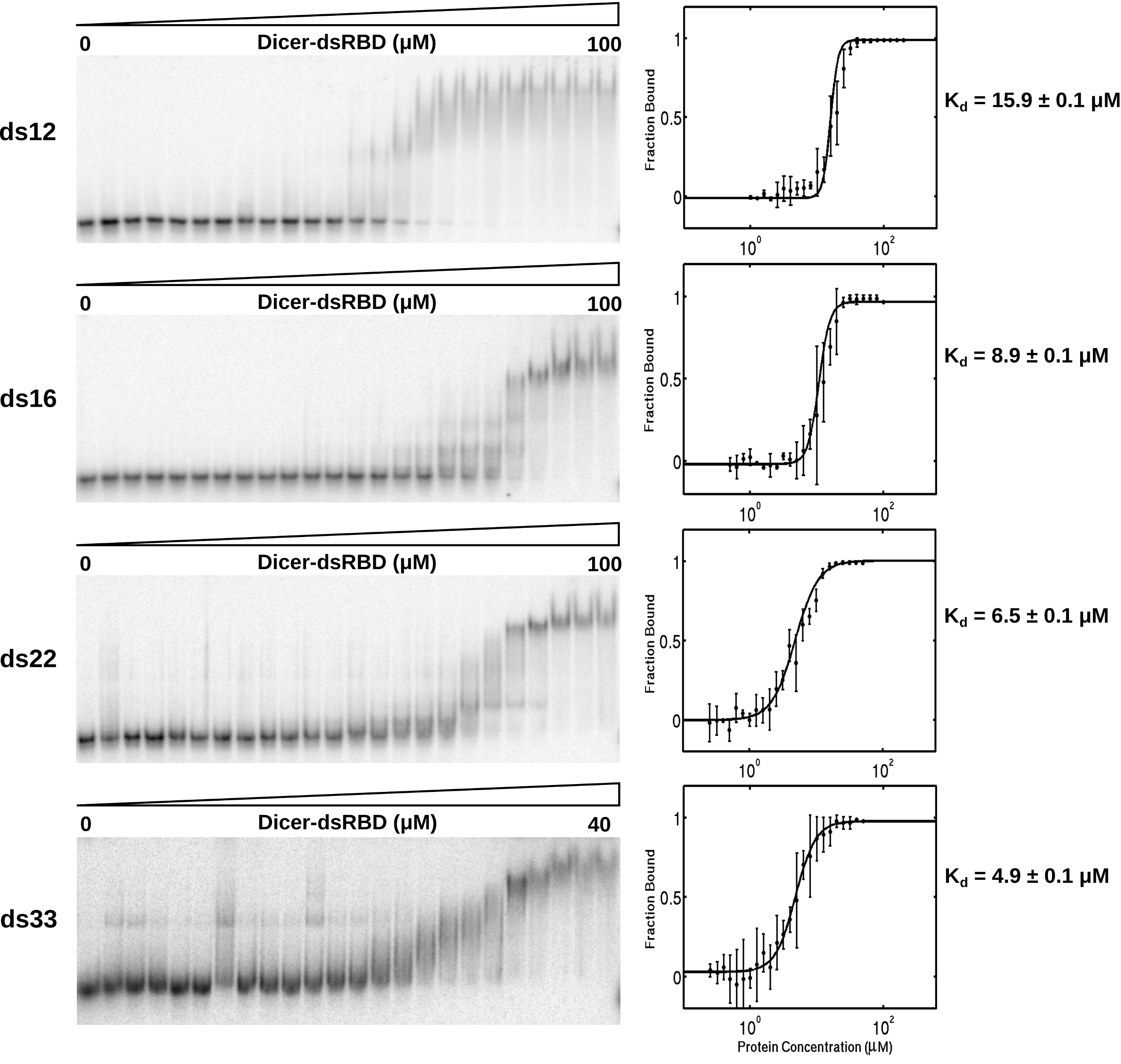

Supplement: Figure S2 — Representative EMSAs of Dicer-dsRBD binding to dsRNA Duplexes. EMSA of Dicer-dsRBD binding to the indicated dsRNA constructs is shown as a representative gel (left) and the fit of fraction bound vs Dicer concentration (right). Best fit lines are to a generalized Hill model and the Kd indicated results from the fitting procedure, as described in the main text. All Watson-Crick duplex constructs used for analysis, but for which a representative gel did not appear in the main text, are represented here. (TIF) [file pone.0051829.s002.tif]

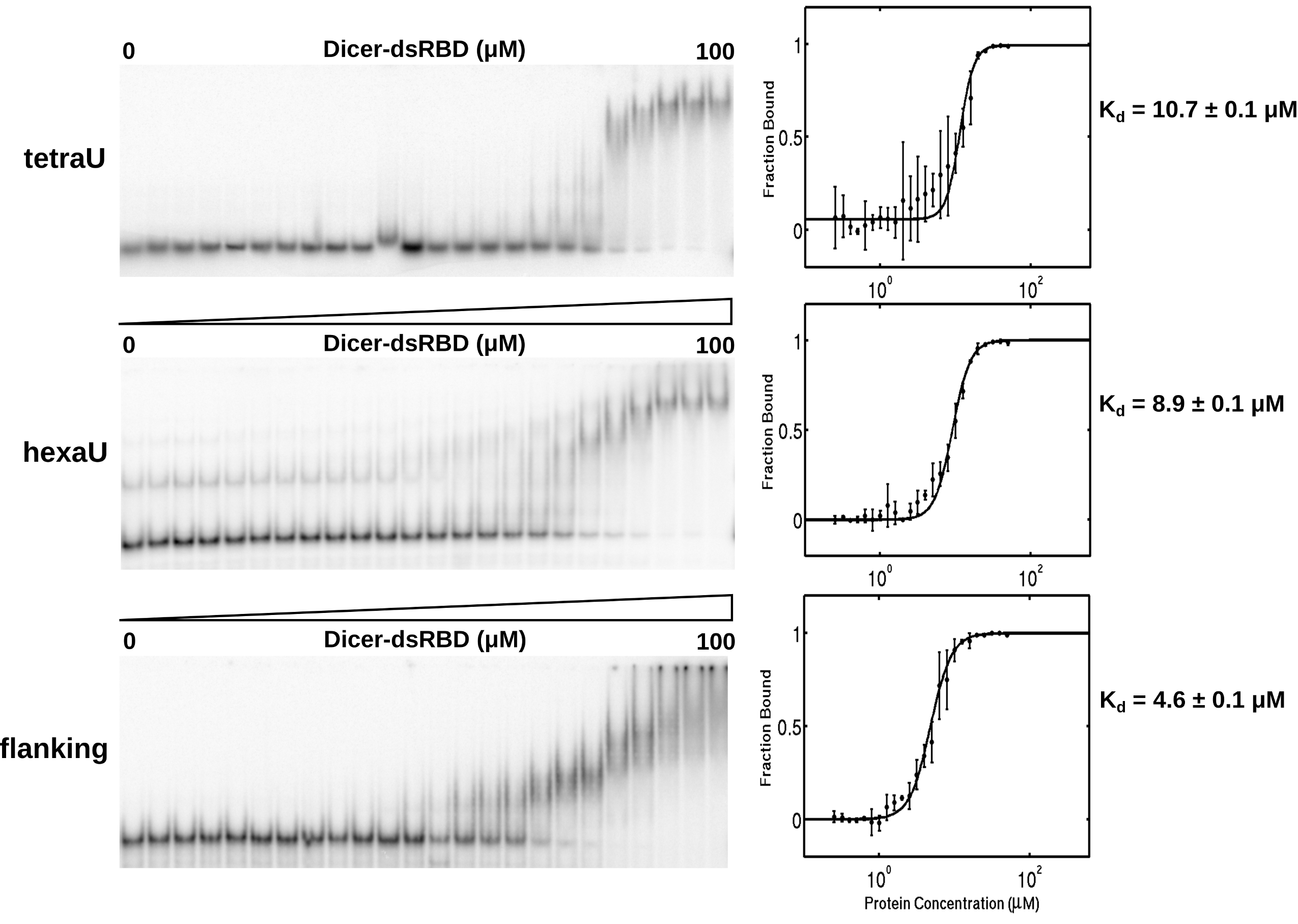

Supplement: Figure S3 — Representative EMSAs of Dicer-dsRBD binding to Loop-terminated dsRNA Duplexes. EMSA of Dicer-dsRBD binding to the indicated dsRNA constructs is shown as a representative gel (left) and the fit of fraction bound vs Dicer concentration (right). Best fit lines are to a generalized Hill model and the Kd indicated results from the fitting procedure, as described in the main text. All loop and tail constructs used for analysis, but for which a representative gel did not appear in the main text, are represented here. (TIF) [file pone.0051829.s003.tif]
