# Supplementary material for: The Role of Human Dicer-dsRBD in Processing Small Regulatory RNAs
Source: PLoS One. 2012 Dec 13;7(12):e51829. doi: 10.1371/journal.pone.0051829 (PMC3521659; doi:10.1371/journal.pone.0051829)
Supplement: Table S1 — Chemical shifts (ppm) from the backbone assignment of Dicer-dsRBD. (DOCX) [file pone.0051829.s004.docx]

**Table S1:** Chemical shifts (ppm) from the backbone assignment of Dicer-dsRBD.

| **Residue** | **N** | **H** | **CO** | **CA** | **CB** |
| --- | --- | --- | --- | --- | --- |
| P1850 | --- | --- | 177.2 | 63.24 | 32.13 |
| R1851 | 120.8 | 8.59 | 176.9 | 57.10 | 29.99 |
| S1852 | 114.4 | 7.99 | 174.4 | 62.23 | 69.59 |
| P1853 | --- | --- | 177.8 | 64.70 | 31.95 |
| V1854 | 117.0 | 7.45 | 177.1 | 67.05 | 31.80 |
| R1855 | 118.0 | 7.10 | 178.5 | 58.70 | 29.48 |
| E1856 | 118.5 | 8.20 | 178.8 | 59.76 | 29.59 |
| L1857 | 119.3 | 8.18 | 177.8 | 58.06 | 41.54 |
| L1858 | 118.1 | 8.11 | 178.9 | 57.06 | 41.24 |
| E1859 | 117.6 | 7.84 | 178.5 | 58.38 | 29.58 |
| M1860 | 118.0 | 7.42 | 176.5 | 57.98 | 34.82 |
| E1861 | 118.0 | 8.05 | 173.2 | 58.17 | 29.20 |
| P1862 | --- | --- | 179.9 | 65.66 | 31.77 |
| E1863 | 114.4 | 8.59 | 177.5 | 55.82 | 30.69 |
| T1864 | 107.5 | 7.41 | 173.6 | 61.08 | 69.74 |
| A1865 | 123.8 | 7.55 | 175.8 | 51.07 | 19.36 |
| K1866 | 122.4 | 8.69 | 175.6 | 54.47 | 34.67 |
| F1867 | 125.2 | 9.24 | 175.9 | 57.99 | 40.48 |
| S1868 | 121.3 | 9.10 | 172.1 | 57.91 | 63.38 |
| P1869 | --- | --- | 176.7 | 62.57 | 32.02 |
| A1870 | 124.0 | 8.56 | 177.3 | 53.05 | 19.70 |
| E1871 | 122.3 | 8.67 | 175.1 | 54.36 | 32.75 |
| R1872 | 124.1 | 8.68 | 177.3 | 55.37 | 31.15 |
| T1873 | 115.6 | 8.51 | 177.0 | 60.76 | 70.81 |
| Y1874 | --- | --- | 175.7 | 59.87 | 37.50 |
| D1875 | 116.2 | 7.94 | 176.3 | 52.33 | 38.85 |
| G1876 | 107.3 | 7.82 | 174.8 | 46.23 | --- |
| K1877 | 119.7 | 7.64 | 174.5 | 55.14 | 33.50 |
| V1878 | 119.0 | 8.61 | 173.5 | 61.48 | 33.64 |
| R1879 | 125.2 | 9.09 | 175.0 | 53.52 | 33.88 |
| V1880 | 124.5 | 8.84 | 173.5 | 61.13 | 36.05 |
| T1881 | 121.3 | 8.56 | 173.6 | 60.95 | 70.88 |
| V1882 | 124.9 | 9.41 | 172.4 | 58.08 | 35.23 |
| E1883 | 129.6 | 8.63 | 175.3 | 54.03 | 31.84 |
| V1884 | 126.9 | 8.90 | 176.1 | 60.48 | 31.87 |
| V1885 | 129.0 | 8.72 | 177.0 | 65.88 | 31.70 |
| G1886 | 115.5 | 8.89 | 174.7 | 45.30 | --- |
| K1887 | 119.0 | 8.63 | 176.0 | 56.73 | 35.14 |
| G1888 | 106.7 | 8.02 | 170.5 | 45.21 | --- |
| K1889 | 117.4 | 7.47 | 175.3 | 54.89 | 35.32 |
| F1890 | 121.4 | 8.94 | 174.0 | 56.59 | 41.96 |
| K1891 | 123.0 | 8.81 | 175.5 | 55.59 | 35.67 |
| G1892 | 109.2 | 8.96 | 171.2 | 44.31 | --- |
| V1893 | 119.7 | 8.10 | 175.6 | 59.59 | 35.58 |
| G1894 | 108.6 | 8.80 | 173.3 | 45.15 | --- |
| R1895 | 118.7 | 7.98 | 176.7 | 62.17 | 32.76 |
| S1896 | --- | --- | --- | --- | --- |
| Y1897 | --- | --- | 175.9 | 61.32 | 38.02 |
| R1898 | 117.8 | 8.63 | 179.1 | 59.76 | 29.94 |
| I1899 | 119.5 | 7.33 | 178.2 | 64.23 | 38.70 |
| A1900 | 124.2 | 8.17 | 178.6 | 55.44 | 18.89 |
| K1901 | 117.9 | 8.60 | 177.3 | 60.06 | 32.67 |
| S1902 | 113.6 | 7.41 | 176.9 | 62.86 | 62.00 |
| A1903 | 123.7 | 8.15 | 180.5 | --- | 18.06 |
| A1904 | 123.3 | 8.39 | 178.8 | 55.25 | 17.80 |
| A1905 | 118.0 | 8.15 | 178.6 | 55.21 | 18.49 |
| R1906 | 117.5 | 8.60 | 178.8 | 60.07 | 30.09 |
| R1907 | 119.7 | 7.71 | 178.6 | 59.28 | 29.73 |
| A1908 | 122.8 | 7.79 | 179.0 | 54.07 | 19.13 |
| L1909 | 117.3 | 8.36 | 178.5 | 58.05 | 42.45 |
| R1910 | 116.9 | 7.56 | 178.7 | 58.92 | 29.87 |
| S1911 | 112.8 | 7.07 | 177.7 | 61.24 | 63.91 |
| L1912 | 120.3 | 8.24 | 179.1 | 57.09 | 41.82 |
| K1913 | 117.1 | 7.93 | 176.7 | 57.11 | 32.47 |
| A1914 | 122.2 | 7.47 | 177.7 | 53.23 | 19.00 |
| N1915 | 116.2 | 8.09 | 174.7 | 53.14 | 38.83 |
| Q1916 | 120.8 | 7.96 | 173.8 | 53.68 | 28.91 |
| P1917 | --- | --- | 176.7 | 62.99 | 32.08 |
| Q1918 | 121.0- | 8.47 | 175.8 | 55.65 | 29.41 |
| V1919 | 122.9 | 8.17 | 174.4 | 59.81 | 32.56 |
| P1920 | --- | --- | 176.6 | 63.03 | 32.20 |
| N1921 | 119.1 | 8.51 | 174.3 | 53.45 | 38.84 |
| S1922 | 120.9 | 7.82 | 178.5 | 59.97 | 64.86 |
